# Specific Appetite for Carotenoids in a Colorful Bird

**DOI:** 10.1371/journal.pone.0010716

**Published:** 2010-05-19

**Authors:** Juan Carlos Senar, Anders Pape Møller, Iker Ruiz, Juan José Negro, Juli Broggi, Esa Hohtola

**Affiliations:** 1 Evolutionary and Behavioural Ecology Associate Research Unit – CSIC, Museu Ciencies Naturals Barcelona, Barcelona, Spain; 2 Laboratoire d'Ecologie, Systématique et Evolution, CNRS UMR 8079, Université Paris-Sud, Orsay, France; 3 Center for Advanced Study, Oslo, Norway; 4 Department of Evolutionary Ecology, Estación Biológica de Doñana-CSIC, Sevilla, Spain; 5 Department of Biology, University of Oulu, Oulu, Finland; University of Oxford, United Kingdom

## Abstract

**Background:**

Since carotenoids have physiological functions necessary for maintaining health, individuals should be selected to actively seek and develop a specific appetite for these compounds.

**Methodology/Principal Findings:**

Great tits *Parus major* in a diet choice experiment, both in captivity and the field, preferred carotenoid-enriched diets to control diets. The food items did not differ in any other aspects measured besides carotenoid content.

**Conclusions/Significance:**

Specific appetite for carotenoids is here demonstrated for the first time, placing these compounds on a par with essential nutrients as sodium or calcium.

## Introduction

Mineral and nutrient appetite is defined as the motivation to seek or choose specific mineral/nutrient-containing items [1]. An specific appetite has been shown, in many different animal species, for sodium [2], calcium [1] and even amino acids [3]. Such “nutritional wisdom” allows animals to regulate their diet choice to satisfy their physiological needs [1]. Carotenoids are involved in physiological processes fundamental for maintenance of health. For example, they are precursors of vitamin A and they are immuno-stimulants [4], [5]. Since carotenoids cannot be synthesised by animals and must be acquired from food, specific appetite for carotenoids should be selectively favored. This should be even more strongly selected in colorful carotenoid-based bird species, in which additional allocation of these compounds for feather pigmentation demands an even larger consumption of carotenoids. Hence, colorful species should especially evolve a specific capacity to seek out food with high levels of carotenoids. Whether this ability exists remains largely unknown.

Here we show that great tits *Parus major*, a small passerine bird with carotenoid-based plumage coloration [6], exhibited a preference for carotenoid-enriched diet.

## Results

**Figure 1:**
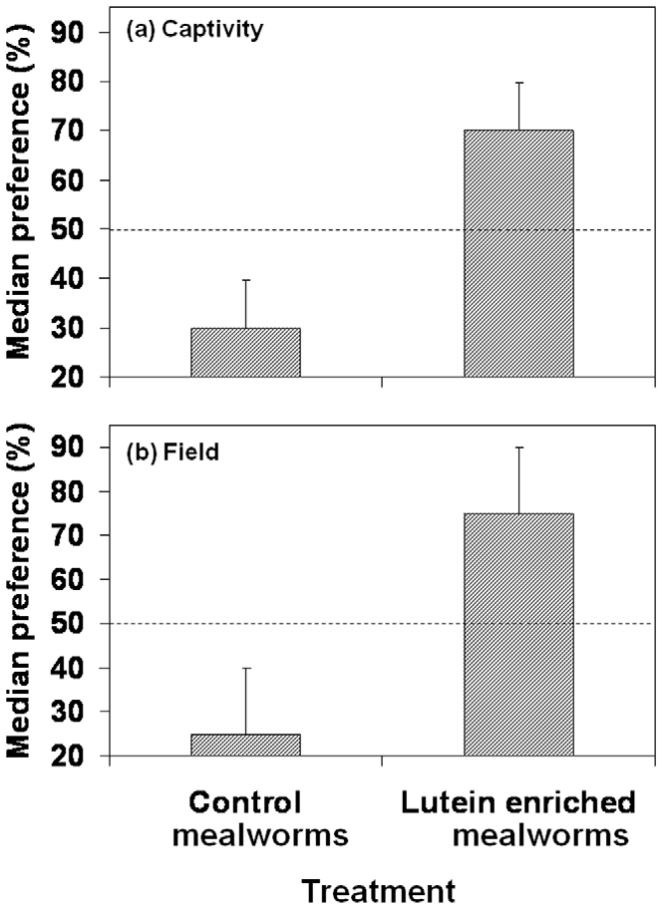
Food choice experiments. Results from the food choice experiments in which great tits were given a choice between two feeders, either containing mealworms with high or low carotenoid content (see Table 1). Results are expressed as the median percentage (±95% confidence interval) of times that the test individual fed from either control or carotenoid enriched mealworms. In the captivity experiment (n = 27 individuals)(a), the carotenoid-enriched mealworms were reared on corn flour, which has a very high content of both lutein and zeaxanthin [18]. Control mealworms were fed with white common wheat flour, which contains low quantities of carotenoids [19] (see also table 1). In the captivity experiment, birds were offered one carotenoid-enriched and one control mealworm, the experiment being repeated for 10 times in different days. In the field experiment, with free ranging wild birds (n = 34 individuals)(b), experimental mealworms were injected with 10 µl of 1.8% lutein and 0.2% zeaxanthin (Kemin Foods, FloraGlo 20% #80447), and control mealworms were injected with 1 µl lutein and zeaxanthin and 9 µl sterile water. In that experiment, individuals were tested only once, and had to choose from 5 experimental and 5 control mealworms.

In captivity choice tests great tits preferentially chose those mealworms (larvae of *Tenebrio molitor*) that had been experimentally enriched with carotenoids over control mealworms (Wilcoxon signed rank test, *T* = 66.5, p<0.05) (Figure 1a), despite them not differing in appearance or any other nutritional aspect than carotenoid content (Table 1).

**Table 1:** Variation in composition and appearance of mealworms.

Variable	Wheat mealworms Mean ± SE	Corn mealworms Mean ± SE	*F*	d.f.	*P*
**Carotenoid content** (µg/g)	0.004±0.018	0.070±0.016	7.66	1,10	<0.02
**Body composition**					
Protein (mg/g –dry mass)	583.39±4.90	575.99±23.43	1.20	1,8	0.31
Fat (mg/g –dry mass)	387.24±4.63	392.62±22.99	0.41	1,8	0.54
**Colour**					
Brightness (%)	46.22±0.83	46.57±0.83	0.09	1,18	0.77
Chroma (%)	21.72±0.48	22.10±0.48	0.31	1,18	0.58
Hue (°)	66.58±0.99	66.93±0.99	0.06	1,18	0.81
UV (%)	3.75±0.49	3.35±0.49	0.33	1,18	0.57
**Size**					
Mass (g)	0.14±0.01	0.13±0.01	0.71	1,18	0.41
Length (mm)	27.0±0.44	26.5±0.44	0.65	1,18	0.43

Variation in composition and appearance of mealworms fed on wheat and corn flour. The two kinds of mealworm differed significantly in carotenoid content, but not in protein or fat composition, or appearance (MANOVA analysis).

In field tests, great tits also significantly chose mealworms with experimentally high levels of carotenoids over controls (Figure 1b) (Wilcoxon matched-pairs test, *T* = 248, *P*<0.001). While there was no significant difference from the null expectation for the first mealworm chosen (56% choosing the high carotenoid treatment; log-likelihood ratio test, *G* = 0.48, d.f. = 1, *P* = 0.85), this preference subsequently increased to 88% for mealworms with high carotenoid levels (*G* = 22.50, d.f. = 1, *P*<0.001). Thus, there was evidence for an increase in preference over time from the first to all later choices combined (*G* = 16.84, d.f. = 1, *P*<0.001). There was no significant effect of sex or age of great tits on the proportion of mealworms taken from the high carotenoid treatment (sex: *F* = 2.55, d.f. = 1, 32, *P* = 0.12; age: *F* = 0.02, d.f. = 1, 32, *P* = 0.90).

## Discussion

Certain animals have foraging preferences for specific colors (e.g. red), that may be related to selection for carotenoid-rich food [7]–[10]. In our experiments, the carotenoid-rich and -poor mealworms did not differ with respect to color, size or nutritional value (as reflected by protein and fat content), yet the birds showed a strong preference for the carotenoid enriched prey type. This preference was remarkable for the captive great tits, since carotenoid concentration in the mealworms feeding on the carotenoid-rich cornmeal was very low, compared to concentration in insects in the wild [11]–[13]. Any preference could in these captive birds be enhanced because of the poor carotenoid supply in their diet.

Although we do not have specific information on the underlying mechanism for the carotenoid preference, the lack of differences in appearance for mealworms used in the different treatments, makes us suggest that the preference may be based on olfaction or taste. There are no known olfactory or gustatory receptors for carotenoids in any animal. However, the volatile degradation products of carotenoids could potentially be readily tasted and smelled. However, we have to emphasize that although in the captivity experiment there was room for a learning process or a post-ingestive physiological feedback mechanism (e.g. hormone interactions or health enhancement by ingested carotenoids that makes birds 'feel good'), since birds were trained to feed from the different mealworms previously to our experiment, great tits in the field tests made their choice of carotenoid enriched mealworms with no intervening training process. This strongly suggests a true specific appetite for carotenoids.

In many different species, carotenoids are fundamental not only for maintenance of health but also for signaling individual quality [14]. Demonstration of specific appetite for carotenoids is highly relevant to theories of visual signaling because it implies that feeding individuals can preferentially detect and choose carotenoid rich food. This novel finding is promising to further investigate signal content.

## Materials and Methods

### Study species

The great tit is a small passerine bird broadly distributed in the Paleartic [15]. The species has a conspicuous yellow breast and belly; this yellow coloration mainly being caused by the presence of lutein and zeaxanthin [6], [12], [13]. The estimated carotenoid body content in the species is 92 µg [16].

### Experimental approach with captive birds

Great tits (N = 27 males) were captured with funnel traps [17] in a forested area in the vicinity of Barcelona, Spain, and transported to nearby facilities of Museu de Ciències Naturals where experimental trials took place. Birds were kept singly in 2 m^3^ cages with water *ad-libitum* and a nest box for roosting for a period of 20 days before being tested. Birds were trained for two weeks previously to the experiment, to eat from two feeders; one, containing carotenoid-enriched mealworms (larvae of *Tenebrio molitor*) and another containing control mealworms. The two feeders were both always present. Mealworms were fed with either corn (carotenoid-rich), or refined wheat flour (control) that differ greatly in carotenoid content, corn flour having a higher content of both lutein and zeaxanthin [18], [19]. This was verified by our own analyses (see below). Feeder position and content was fixed within each cage/individual and varied randomly among individuals. Experimental trials consisted of (simultaneously) offering each individual one carotenoid-enriched and one control mealworm in the corresponding feeders, and recording the feeder at which the tit first foraged. Each individual was tested once per day for 10 days.

Great tits were captured and maintained in captivity with a special permission from the Catalan Government, Direcció General del Medi Natural, Generalitat de Catalunya. Experiments were conducted according to Catalan guidelines for the use of animals in research. The cages used were large enough (see before) to guarantee Great tit welfare. All the cages contained a nest box for roosting and resting, and food and water were available *ad libitum*. Since animals were housed individually, birds were not subjected to any social stress. Since experimentation consisted only of diet choice trials, and food provided consisted only of mealworms, approval by a special committee was not necessary.

### Experimental approach with free ranging birds

Great tits were captured in mist nets near commercial bird feeders consisting of fat and seeds during December 2008– February 2009 in Orsay, France, and they were subsequently provided with color rings for identification. The food preference test was also based on mealworms differing in carotenoid content. Mealworms were kept on a diet of wheat flour with low carotenoid content until use. Following capture of great tits, pairs of 5 cm diameter cups for food with a distance between cups of 10 cm were provided in the same sites as where mist nets were used, with one randomly chosen cup holding either 1). five mealworms each injected in the hemocoel with 10 µl of 1.8% lutein and 0.2% zeaxanthin (Kemin Foods, FloraGlo 20% #80447), or 2). five mealworms injected with 1 µl lutein and zeaxanthin (hereafter carotenoids) and 9 µl sterile water. The concentrations of carotenoids in mealworms were thus 1.43 and 0.14 µg/g, respectively. The amounts injected were so small that they remained inside the mealworms, and great care was taken to ensure that the liquid was not visible on the outside. These two treatments were chosen to assure that carotenoids were present in both treatments, and that a potential preference would relate to the amount of carotenoids rather than mere presence or absence of carotenoids. A total of 10 µl carotenoids in one mealworm amounted to 0.2% of estimated body content (92 µg) [16], or 8.4% of total stored carotenoids in the liver of a great tit [concentration in liver was 3.80 µg/g for a liver weighing on average 0.63 g, *N* = 11 great tits; A. P. Møller, J. Erritzøe and F. Karadas unpublished information]. This treatment assured that carotenoid-rich and control mealworms differed only in carotenoid content.

Using a pair of binoculars at a distance of 30 m APM recorded for each individual great tit visiting the choice apparatus the number and the order of mealworms taken from each of the two cups during a period of 15 min, or until the bird had ingested all (5) mealworms from one of the cups. A total of 34 great tits were tested during these trials. Each individual was tested only once. All birds ate from both feeders.

### Analysis of mealworm coloration for the captivity experiment

The coloration of the two types of mealworm was recorded using a Minolta CM-2600d spectrophotometer, that measures from 360 to 700 nm and provides values of brightness, chroma and hue (LCH) using the standard software provided by the instrument [20]. However, algorithms to calculate LCH variables refer only to the 400–700 nm range (i.e. that visible to the human eye) and omit the UV region that is visible to birds. For this reason, and given that the maximum peak of absorbance of the fourth cone of vision in the UV range in the closely related blue tit (*Cyanistes caeruleus*) has λ = 371 nm [21], we also included reflectance at 370 nm to take UV reflectance into account. We measured all spectra with reference to a white standard (WS-1, Diffuse Reflectance Standard) (reflectivity over 98%) and with reference to a dark spectrum to avoid external light contamination.

We measured coloration on the dorsal area and flanks of the mealworms used in the aviary experiment. Three measurements were obtained from each mealworm on different random areas of the body, and values were averaged. We ensured a good measurement by using a mesh of 3 mm and by gently pressing on the mealworms.

### Analysis of carotenoid content of mealworms for the captivity experiment

Both types of mealworms were ground up to obtain a homogeneous mixture. For carotenoid extraction, one gram of each sample was weighed in triplicate and placed into falcon tubes containing 25 ml acetone. Suspensions were subsequently re-ground in ultra-turrax and vacuum filtered. Extraction procedures were repeated several times until filtrates were completely colourless.

Extracts were then combined in decanting flasks and 25 ml ethyl ether were added. We homogenized solutions and added 50 ml of a 10% aqueous NaCl solution. The colorless hypophases were discarded and the epiphases repeatedly washed with the NaCl solution. Epiphases were filtered through beds of anhydrous sodium sulfate and placed in rotary-evaporator flasks. Solvents were evaporated and pigment concentrates recovered from flasks with small additions of HPLC grade acetone to a final volume of 10 ml. Aliquots of 0.5 ml from different pigment solutions were filtered through syringe filters with 0.2 µm of pore diameter for subsequent injection in an HPLC system.

The separation system used was described by Mínguez-Mosquera and Hornero-Mendez [22], modified by Negro et al. [23]. We used a reverse-phase column (Spherisorb ODS2) of 25 cm in length, 0.46 cm internal diameter, and with a particle size of 5 µm. Separation was performed using an acetone-water binary gradient with a flow of 1.5 ml min^−1^. The volume of sample injected was 20 µl, and detection was performed at 450 nm using a fixed-wave UV-visible detector.

Carotenoid pigments were identified by comparing their retention time and spectral data under the elution conditions with those obtained using pure standards [22], [24]–[26]. Carotenoid concentration was determined by comparing the area of each peak in the chromatogram with areas of the calibration curve obtained using pure standards. Quantification results are presented as means of three independent injections.

### Analysis of protein and fat content of mealworms for the captivity experiment

One gram of mealworms (in 5 replicates) were placed in pre-weighed extraction thimbles and dried for 48 h at 80°C. They were cooled in an exsiccator and weighed on an analytical balance after which the thimbles were placed in a Soxhlet-apparatus. Neutral fat was extracted using a mixture of petroleum ether and chloroform (95/5 v/v) overnight. The mass decrease after drying at 40°C was taken as the amount of neutral fat. The remaining mass can be taken to consist of protein and ash. Ash content was determined by incinerating a subsample of the residue in a pre-weighed crucible at 600°C for 24 h. The method is the standard according to Blem [27].

### Statistical analyses

In the diet choice preference experiments with captive birds, we measured the percentage of times that test individuals had fed from the carotenoid enriched mealworm along 10 tests. This percentage was used as a measure of preference (or avoidance). We analysed the data with a Wilcoxon signed-rank test [28]. Our null hypothesis was that 50% of the times tits should start feeding from either of the two experimental mealworms, and a significant deviation from 50% indicated a significant preference.

In the diet choice preference experiments with free ranging wild birds, we compared for each individual (matched-pairs test) the number of carotenoid-enriched and control mealworms that it had ingested. For the analysis of a preference for the first mealworm chosen and for the subsequent selections, we used the log-likelihood ratio test, with a null hypothesis of 50% ingestion of carotenoid enriched mealworms.
